# Love Influences Reproductive Success in Humans

**DOI:** 10.3389/fpsyg.2017.01922

**Published:** 2017-11-21

**Authors:** Piotr Sorokowski, Agnieszka Sorokowska, Marina Butovskaya, Maciej Karwowski, Agata Groyecka, Bogdan Wojciszke, Bogusław Pawłowski

**Affiliations:** ^1^Institute of Psychology, Faculty of Historical and Pedagogical Sciences, University of Wrocław, Wrocław, Poland; ^2^Department of Psychotherapy and Psychosomatic Medicine, Technische Universität Dresden, Dresden, Germany; ^3^Institute of Ethnology and Anthropology, Russian Academy of Sciences, Moscow, Russia; ^4^Sopot Faculty of Psychology, University of Social Sciences and Humanities, Warsaw, Poland; ^5^Department of Human Biology, University of Wrocław, Wrocław, Poland

**Keywords:** romantic (love), sexual selection, number of children, Hadza, commitment, passion, human evolution, hunter-gatherers

## Abstract

As love seems to be universal, researchers have attempted to find its biological basis. However, no studies till date have shown its direct association with reproductive success, which is broadly known to be a good measure of fitness. Here, we show links between love, as defined by the Sternberg Triangular Theory of Love, and reproductive success among the Hadza—traditional hunter-gatherer population. We found that commitment and reproductive success were positively and consistently related in both sexes, with number of children showing negative and positive associations with intimacy and passion, respectively, only among women. Our study may shed new light on the meaning of love in humans' evolutionary past, especially in traditional hunter-gatherer societies in which individuals, not their parents, were responsible for partner choice. We suggest that passion and commitment may be the key factors that increase fitness, and therefore, that selection promoted love in human evolution. However, further studies in this area are recommended.

## Introduction

The phenomenon of love, encompassing cognitive, emotional, and social components (Fisher, 2004), is broadly discussed in the scientific literature and popular culture (Dukes et al., 2003; Cox and Fisher, 2009), underscoring its importance in people's everyday lives. The universality of love (Jankowiak and Fischer, 1992; Jankowiak, 1995, 2008) indicates that it has a biological basis (Bartels and Zeki, 2004; Fisher et al., 2005, 2006; Young, 2009; Carter and Porges, 2012). Researchers have attempted to isolate and identify neural (Bartels and Zeki, 2004; Fisher et al., 2005; Young, 2009), hormonal (Fisher, 2004; Carter and Porges, 2012), and genetic (Walum et al., 2008) components underlying this seemingly uniquely human phenomenon. The main unanswered questions so far are why love was promoted by human evolution and how it is related to biological fitness (Sorokowski et al., 2017).

Due to the high level of subjectivity of the phenomenon, studying about love is not easy. Among many psychological theories of love (see Hatfield et al., 2012), the Triangular Theory of Love by Sternberg (1986) is one of the most popular ones; it is also broadly used in empirical research (e.g., Aron and Henkemeyer, 1995; Sumter et al., 2013; Sabiniewicz et al., 2017). According to Sternberg, love is formed as a triangle with the following key constructs as vertices: intimacy, passion, and commitment (Sternberg, 1986) Passion pertains to romance, physical attraction, and sexual intercourse. The intimacy component refers to emotional aspects of love: closeness and warmth in loving relationships. It is primarily derived from the emotional involvement in the relationship. Finally, commitment (also referred to as *decision*) pertains to cognitive decisions about starting and maintaining a long-term relationship. Sternberg (1986) suggested that the construct of love is dynamic and hypothesized that commitment, although not rapidly, increases with relationship duration, whereas passion and intimacy gradually decrease; commitment is also described as relatively stable in time, especially compared to passion (Sternberg, 1986). Empirical research confirmed that the level of commitment is higher in couples in more serious relationships (e.g., marriages) and that it increases in time (Acker and Davis, 1992). According to Solomon (1980), whose works served as a basis for Sternberg's theory, the decrease of passion with increasing relationship duration can be due to the lack of balance in partner's arousal in further stages of a relationship. Several studies confirmed this decline (Acker and Davis, 1992; Tucker and Aron, 1993; Sprecher and Regan, 1998; Lemieux and Hale, 2002; Ahmetoglu et al., 2010). More recent study (Schröder and Schmiedeberg, 2015) has identified pregnancy and time, when couple has small children, as a temporary period of decreased sexual activity, hence indicating a U-shape trajectory of passion. Studies revealed mixed results in terms of shifts in intimacy (Acker and Davis, 1992; Lemieux and Hale, 2002), although shifts in passion and intimacy may be highly related to one another (Baumeister and Bratslavsky, 1999).

All three Sternberg's love components could be important in the context of human reproductive success and might be considered as biological adaptations, but they play differential roles in mating. Passion initiates a relationship, motivating sexual interest and proximity seeking (Leridon, 1993) that are necessary to conceive a child. Sexual desire increases in response to cues and markers of fertility and reproductive status (e.g., physical attractiveness), and hence, it is highly important for reproduction (Buss, 2006; Gonzaga et al., 2006). Indeed, Hopcroft (2006) found a positive correlation between the number of children and frequency of sexual intercourses (and thus likely higher passion). In addition, passion breeds partner idealization (Gagné and Lydon, 2004) and derogation of potential alternative mates (Johnson and Rusbult, 1989), both of which contribute to monogamous character of a relationship. Intimacy might fulfill a role in sustaining long-term relationships by promoting connection between partners and mutual long-term plans (Sternberg, 1986; Diamond, 2003). Increased proximity induced and sexual desire provoked by passion promote contact and allow commitment to grow (e.g., Gonzaga et al., 2006), and commitment again can suppress thoughts of attractive alternative mates (Gonzaga et al., 2008). Consequently, commitment and intimacy, described jointly as “romantic love” (Sternberg, 1986), were found to promote biparental support of the offspring (Hill and Hurtado, 1996; Marlowe, 2003; Gonzaga et al., 2006). For example, among the Hadza, having an infant decreases a mother's efficiency in foraging. This deficit is compensated by her husband's higher activity. Therefore, male provisioning could be a notable aspect of pair bonding (Marlowe, 2003). In this context, love might have been particularly important in our evolutionary past, as presence of both parents and/or greater food resources provided to offspring during critical periods of a child development might increase offspring survival rate (Hill and Hurtado, 1996; Marlowe, 2003; Winking, 2006). Overall, it can be suggested that love, as a function of all the three described components, should increase reproductive success through natural and sexual selection. This hypothesis has been presented many times (e.g., Campell and Ellis, 2005; Buss, 2006; Pillsworth and Haselton, 2006), but it has never been empirically tested among humans.

Many factors, like effective contraception or family planning, disturb the potential relationship between love and number of children, making it very difficult to study the relationship of these two elements in contemporary Western societies. Therefore, we decided to study potential love-fitness association among the Hadza (Tanzania, Africa), a hunter-gatherer society, whose lifestyle is in many ways similar to that of our ancestors (Apicella et al., 2012). To study love in this tribe, we used the three components of the Triangular Theory of Love by Sternberg (Sternberg, 1986, 1997): intimacy (closeness and warmth), passion (physical attraction and sexual activity), and commitment (Sternberg, 1986, 1997).

## Materials and methods

The study was conducted according to the principles of the Declaration of Helsinki. The study protocol and consent procedure received ethical approval from the Institutional Review Board (IRB) and the Tanzania Commission for Science and Technology (COSTECH). Because the population studied was illiterate, written consent could not be obtained, and therefore, the participants gave verbal consent and were told that their participation was voluntary and that they could quit at any time. Obtaining verbal consent was also approved by the IRB.

### Participants

The data were collected among the Hadza of Tanzania, a traditional hunter-gatherer tribe (Marlowe, 2010; Jones, 2016). The Hadza number included approximately 1,000–1,300 individuals (Marlowe, 2010). They are monogamous, with only about 4% of men having two wives, and virtually all Hadza adults are married (Marlowe, 2010). Marriages are typically not arranged, and divorce is common (Marlowe, 2010). In total, 159 people (83 men and 76 women) aged 16–70 years participated in this study; all of them were married.

### Procedure

Data were collected during individual interviews in Swahili with the help of a local interpreter. Love was studied with Sternberg's Triangular Love Scale (Sternberg, 1997), which was back-translated from English to Swahili. Based on a pilot study on 15 people, we shortened the original scale to 17 items (5, 6, and 6 on intimacy, passion, and commitment, respectively; e.g., “I find myself thinking about __ frequently during the day.” for passion; “I share deeply personal information about myself with __.” for intimacy; “I view my relationship with __ as permanent.” for commitment; for all items see Appendix 1). Based on verbal feedback of the pilot study participants, we removed all items that were rated as unclear and/or difficult to understand by any of the participants. Because our respondents were unfamiliar with Likert-type scales, the response options were simplified to a “yes—to some extent—no” scale. The scales were scored by averaging the items. The alpha coefficients estimated on polychoric correlation matrices were generally acceptable, given short scales (5 or 6 items) and short response scale: intimacy (α = 0.63), passion (α = 0.64), and commitment (α = 0.75).

After answering the love questionnaire, the participants were asked about children born in their current marriage. Participants reported having had 0–12 children (*M* = 2.59; *SD* = 2.38) of whom 0–6 died before reaching reproductive age (14 years) (*M* = 0.44; *SD* = 0.86)—see Table 1 for descriptive statistics and correlations between variables. We also controlled for the participants' weight and height. Because the Hadza are an egalitarian society (Marlowe, 2003) and individuals' economic status is similar, we did not control for this variable. We also controlled for participants' age, and when some participants did not know their exact age, it was estimated by one of the authors (e.g., on the basis of what the individual remembered from history or peer tribe members). We also measured height and weight as control variables, as they are often used as proxies for food availability related with sexual hormones, or direct reproductive success among traditional societies (MacDonald et al., 2009; Rickard et al., 2010; Sorokowski et al., 2013); we used body-mass index (BMI) for control purposes in our analyses.

**Table 1:** Descriptive statistics and correlations between the main variables.

**Variables**	**Min**	**Max**	***M***	***SD***	**Men *M* (*SD*)**	**Women *M* (*SD*)**	**2**	**3**	**4**	**5**	**6**
Children total	0	12	2.59	2.38	2.60 (2.39)	2.57 (2.40)	0.51^***^	−0.02	0.06	0.13	0.26^**^
Age	16	70	36.64	12.43	39.22 (12.74)	33.83 (11.53)	1	−0.11	0.19^*^	0.12	0.25^**^
BMI	14.79	27.38	20.56	1.98	20.58 (1.77)	20.54 (2.20)		1	−0.10	0.03	0.002
Intimacy	1	3	2.54	0.38	2.59 (0.35)	2.50 (0.41)			1	0.43^***^	0.49^***^
Passion	1.17	3	2.56	0.36	2.60 (0.30)	2.53 (0.41)				1	0.57^***^
Commitment	1	3	2.53	0.38	2.56 (0.35)	2.50 (0.42)					1

N = 159. In the case of correlation coefficients Spearman's rho are presented in all cases except the correlations between age and BMI—in this case Pearson's r is given;

* *p < 0.05*,

** *p < 0.01*,

*** *p < 0.001*.

## Results

**Figure 1:**
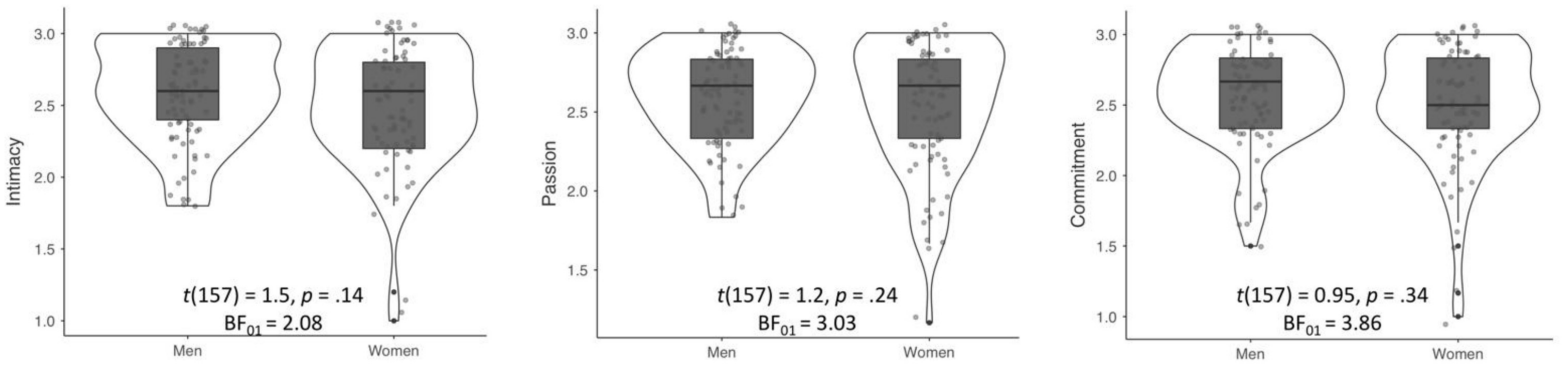
Sex differences in love components.

Descriptive statistics and correlations between variables are presented in Table 1. The level of measured love components—intimacy, passion, and commitment—was similar among men and women; the observed differences were not significant, and Bayes Factor suggests moderate support for the null hypothesis (intensity of intimacy, passion, and commitment is the same across sexes); see also Figure 1. Age was positively related to the level of intimacy (*r* = 0.19, *p* = 0.02) and commitment (*r* = 0.25, *p* = 0.003), yet unrelated to passion (*r* = 0.12, *p* = 0.18). The number of children was linked to commitment (*r* = 0.26, *p* = 0.001).

To examine the effect of each love component on reproductive success, the number of children was regressed onto the love components and control variables (i.e., age, sex, BMI) (Table 2, model A). In addition, we included the *Sex* × *Love Component* interactions in subsequent models to control for possible sex differences in the effects of love components on reproductive success (Table 2, model B). Because the reported number of children was skewed (skewness = 1.33, kurtosis = 1.96), we used Poisson regression. To facilitate interpretation, we also provided β*-* and *R*^2^-values from linear regression models with log-transformed dependent variables.

**Table 2:** Love factors as predictors of total number of children.

	**Children Total**					
	**Model A**			**Model B**		
**Predictors**	***B* (*SE*)**	**Exp *B***	**β**	***B* (*SE*)**	**Exp *B***	**β**
Intercept	0.44(0.22)	1.55	–	0.82(0.07)^***^	2.27	–
Sex	0.13(0.07)	1.14	0.11	0.12(0.07)	1.13	0.11
Age	0.46(0.07)^***^	1.58	0.50	0.46(0.06)^***^	1.58	0.50
BMI	−0.02(0.06)	0.98	–0.01	−0.003(0.05)	1.00	−0.01
Intimacy	−0.04(0.09)	0.96	–0.07	−0.03(0.08)	0.97	−0.09
Passion	0.08(0.11)	1.09	0.05	0.06(0.09)	1.07	0.04
Commitment	0.23(0.11)^*^	1.26	0.19	0.24(0.11)^*^	1.27	0.21
Sex × Intimacy	–	–	–	−0.23(0.08)^**^	0.80	−0.23
Sex × Passion	–	–	–	0.22(0.09)^**^	1.24	0.24
Sex × Commitment	–	–	–	−0.05(0.10)	0.96	−0.05
Deviance (*df*)	205.55(**df** = 118)	189.23 (*df* = 115)				
Model A vs. B	16.3(**df** = 3)^**^					
*R*^2^	0.27	0.30				

*N = 159. Sex coded: Men = −1, Women = 1. All predictors except sex were introduced to the models as z-scored variables. All unstandardized coefficients (β-values with robust standard errors) should be read as the expected increase or decrease (in case of positive and negative coefficient values, respectively) of the dependent variable (i.e., the number of children) in log units for a one-unit increase in predictor. For a more convenient interpretation, we also present results as incident rate ratios (ExpB), showing the percent change in incident rate of number of children related to a unit change in predictors. β- and R^2^-values came from linear regression models with log-transformed dependent variables*.

* *p < 0.05*,

** p < 0.01;

*** *p < 0.001*.

**Figure 2:**
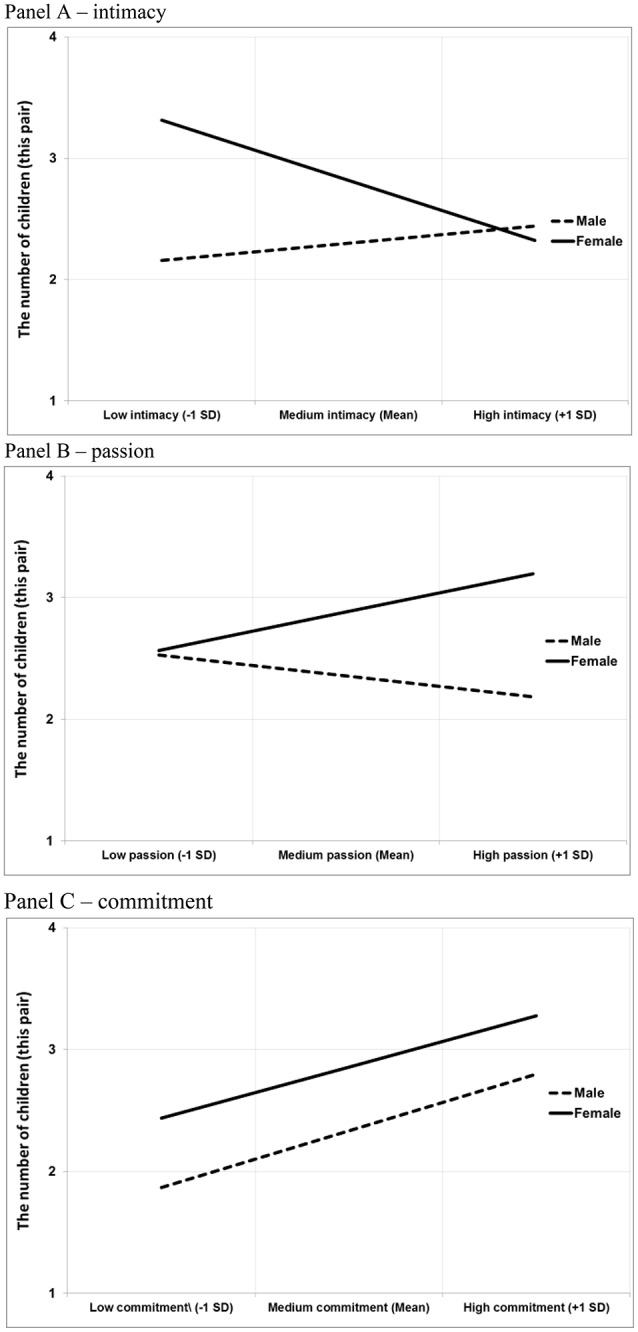
Interactions between sex and love aspects on number of children. **(A)** demonstrates different effects of intimacy on number of children among women and men: the effect is negative and significant among women (*B* = −0.26, *SE* = 0.11, *p* = 0.02), but not among men (*B* = 0.15, *SE* = 0.08, *p* = 0.07). **(B)** shows a positive effect of passion on number of children among women (*B* = 0.24, *SE* = 0.12, *p* = 0.05), but not among men (*B* = −0.16, *SE* = 0.12, *p* = 0.19). **(C)** illustrates positive effects of commitment on number of children; the effect is significant among men (*B* = 0.27, *SE* = 0.12, *p* = 0.02) but not women (*B* = 0.17, *SE* = 0.16; *p* = 0.30).

Commitment was a statistically significant positive predictor of the number of children. We found no interactions with this love component, but we observed significant *Sex* × *Intimacy* and *Sex* × *Passion* interactions. The associations between intimacy and passion on the one hand and the number of children on the other were significant for women (intimacy: *B* = −0.26, *SE* = 0.11, *p* = 0.02, *ExpB* = 0.77; passion: *B* = 0.24, *SE* = 0.12, *p* = 0.05, *ExpB* = 1.27), but not for men (intimacy: *B* = 0.15, *SE* = 0.08, *p* = 0.07, *ExpB* = 1.17; passion: *B* = −0.16, *SE* = 0.12, *p* = 0.19; *ExpB* = 0.86) (see Figure 2)^1^. No significant findings were observed for mortality rate (see Table 3).

**Table 3:** Love factors as predictors of mortality rate.

	**Mortality rate**					
	**Model A**			**Model B**		
**Predictors**	***B* (*SE*)**	**Exp *B***	**β**	***B* (*SE*)**	**Exp *B***	**β**
Intercept	2.41(0.19)^***^	11.15	–	2.38(0.20)^***^	10.85	–
Sex	0.10(0.18)	1.11	0.09	0.10(0.19)	1.10	0.07
Age	0.23(0.17)	1.26	0.25^**^	0.21(0.17)	1.23	0.24^**^
BMI	−0.10(0.20)	0.90	−0.04	−0.11(0.19)	0.90	−0.04
Intimacy	0.02(0.17)	1.02	−0.003	0.04(0.17)	1.05	−0.003
Passion	−0.23(0.18)	0.80	−0.03	−0.18(0.17)	0.83	−0.02
Commitment	0.27(0.18)	1.31	0.12	0.30(0.18)	1.35	0.14
Sex × Intimacy	–	–	–	−0.04(0.17)	0.96	−0.15
Sex × Passion	–	–	–	−0.10(0.17)	0.91	0.04
Sex × Commitment	–	–	–	−0.07(0.19)	0.93	0.009
Deviance (*df*)	3, 963.23(**df** = 118)	3,931.70 (*df* = 115)				
Model A vs. B	31.53(**df** = 3)^***^					
*R*^2^	0.09	0.10				

*Sex coded: Men = −1, Women = 1. Dependent variable (mortality rate) calculated on a 0–100 scale, with 0 = 0%, 100 = 100%. All predictors except sex were introduced to the models as z-scored variables. All unstandardized coefficients (B-values with robust standard errors) should be read as the expected increase or decrease (in case of positive and negative coefficient values, respectively) of the dependent variable (i.e., mortality rate) in log units for a one-unit increase in predictor. For a more convenient interpretation, we also present results as incident rate ratios (ExpB), showing the percent change in incident rate of number of children related to a unit change in predictors. β- and R^2^-values came from linear regression models with log-transformed dependent variables*.

** p < 0.01;

*** *p < 0.001*.

Row data are available at: https://figshare.com/s/6a005c8f6885d6a6b3fd.

## Discussion

Our study provides the first evidence that human love is related to reproductive success. Among the three investigated love components, commitment was positively related to reproductive success for both the sexes. Passion and intimacy were positively and negatively associated with the number of children, respectively, but only for women.

In our study, commitment positively correlated with the number of children for both the sexes. This is quite logical, as commitment helps to maintain stable relationships (Johnson and Rusbult, 1989), and a decision to stay together typically precedes a decision to have children (Ranson, 2008). However, in the case of this love component, it is hard to determine the causality. Theoretically, the number of children might influence the desire to maintain a relationship, and not vice versa, as studies in Western societies show that higher number of children is also associated with lower divorce rate (Cherlin, 2010). Yet, if that was the case, then one could expect that lower commitment would be related to higher child mortality (Hill and Hurtado, 1996; Marlowe, 2003), and we did not observe such an association. However, we conducted our study among individuals who had partners—their level of commitment was, therefore, high enough to keep them together. In addition, the relationship between mortality and father's presence is sometimes questioned (Winking et al., 2011a,b).

Theoretically, from the evolutionary point of view, passion seems to be particularly important in the adaptive context (Fisher, 2004; Gonzaga et al., 2006, 2008). First, it increases the number of sexual encounters (Leridon, 1993; Hopcroft, 2006). Furthermore, it intensifies in response to fertility markers and might, thus, promote mating with individuals of the highest reproductive potential (Buss, 2003). Theoretically, there is also other reason why this love component can be perceived as adaptive. From the evolutionary perspective, reproduction is one of the aims of establishing a relationship. As discussed in the introduction, passion is particularly high in the early stages of a relationship (Sternberg, 1986). Thus, if a couple cannot have children, then partners will realize that quite quickly. They could then decide to remain in a current relationship or search for alternative mates. Interestingly, in our sample, passion was related to number of children only for women. This could mean that only female passion determines the number of sexual encounters, and consequently, reproductive success. Although our research was correlational, causality in this case is likely, as explaining the relationship between passion and reproductive success in terms of the influence of number of children on passion seems implausible. In a broader perspective, it can be hypothesized that love (or its loss) might be more adaptive for women (Perilloux and Buss, 2008). It is likely that due to higher biological investment in parenting (Trivers, 1972), partner idealization (Gagné and Lydon, 2004) is necessary for a woman to decide whether to engage in sexual intercourse (and potential pregnancy). Furthermore, women could be more sensitive than men when selecting fathers for their children and their sexual arousal could be higher in response to genetically optimal partners. For example, Garver-Apgar et al. (2006) found that female (but not male) responsiveness to the current partner negatively correlated with the degree of human leukocyte antigen (HLA) allele-sharing with this partner. Rejection of HLA-similar partner's sexual advances was found to be stronger during the fertile phase of the menstrual cycle. This seems very important in the adaptive context, as HLA-dissimilarity can increase the probability of successful conception and pregnancy, and might give the potential offspring advantage under immune challenge (see Havlicek and Roberts, 2009 for a review). However, it needs to be noted that at this stage of research, all presented hypotheses require further studies.

The observed pattern of results for intimacy differed from our hypothesis—we found an unexpected negative relationship between intimacy and number of children in women. Possibly, women who need to take care of more children have less time to spend with their partners, explaining the decreased level of this love component. Mothers can also satisfy their need for intimacy with close contact with their children. Indeed, studies have shown that the transition to parenthood can interfere with couples' sex life and companionship (Twenge et al., 2003) and that marital relations and parent–child relations are interrelated (Erel and Burman, 1995).

Our results might be analyzed in the context of interrelationships among the love components presented in Sternberg's theory (1986). The data imply that fatuous love (in women) and empty love (in men) drive reproductive success. Fatuous love is a combination of passion and commitment (Sternberg, 1986). In this type of relationship, commitment is based on passion without the stabilizing influence of the intimacy component. Empty love is based exclusively on commitment.

It is yet to be tested and explored why we observed these sex differences among the Hadza (although it needs to be remembered that our analysis of interactions between love components did not yield any significant results). One hypothesis is that temporal dynamics of the love components differ for Hadza men and women, resulting in procreation at different love stages. Sternberg (1986) suggested that in some societies, empty love might be the first stage of a relationship (this is contrary to modern, Western societies where empty love is usually observed during a near-final stage of a relationship). Marital partners (in this case, it is Hadza men) may, thus, make a conscious decision to form a relationship (or to have children with a given woman) and then gradually come to love their partners. This would result in procreation when commitment is increasing, and when passion and intimacy are virtually absent. At the same time, for Hadza women, a decision to marry and to have children with a particular man might depend more on emotional factors, thereby resulting with fatuous love being a driving force behind reproductive success. In future studies, researchers could test whether the assumed dynamics of the three love components are culturally universal and whether the temporal changes and the observed correlations between love components and reproductive success are analogous for men and women all over the world.

It should be mentioned that our research had certain limitations. First, we do not know whether Hadza understand love in a way operationalized by Sternberg (1986) (however, we also cannot be certain that members of Western societies do so). Nevertheless, we intended to analyze whether the concept presented in Western scientific literature as love can be related with reproductive success among a traditional society. Second, it is generally very hard to discuss what love really is and how it should be tested. For example, it can be difficult to distinguish between passion understood as general sexual desire and passion resulting from loving a particular person (for a discussion see Wolf, 1970; Symons, 1979). Third, relationship length could be a better control variable than participants' age; however, it would be very difficult to reliably assess this variable in traditional societies. Furthermore, some Hadza women have a series of long-term relationships with different men. However, this situation resembles the pattern of long-term partnerships in modern Western societies. Finally, the correlational nature of our study seems to be the most important limitation of our research, thereby decreasing the possibility to determine causality for our findings. For example, although it does not sound probable, we cannot rule out a possibility that number of children increases passion among Hadza. Anyway, unlike in the case of animal studies (Ihle et al., 2015), experiments with this regard are not possible, and longitudinal research would be extremely difficult given, among others, the nomadic lifestyle of Hadza.

In summary, the present study on Hadza people may shed new light on the meaning of love in humans' evolutionary past, especially in traditional hunter-gatherer societies in which individuals, not their parents, were responsible for partner choice. We hypothesize that passion and commitment were among the key factors increasing fitness, and therefore that selection might promote love in human evolution. Despite some limitations, our research was the first empirical examination of a very important issue and should stimulate extensive works in this area.

## Author contributions

Conceived and designed the experiments: PS, AS, BP, and BW. Performed the experiments: MB and AG. Analyzed the data: MK. Wrote the paper: PS, AS, AG, MK, BP, MB, and BW.

### Conflict of interest statement

The authors declare that the research was conducted in the absence of any commercial or financial relationships that could be construed as a potential conflict of interest.

## Footnotes

^1^An anonymous reviewer also suggested testing an additional possibility, stemming directly from Sternberg's (1986) theory of love. More specifically, the reviewer proposed that we also include interactions between love components (so *Intimacy* × *Passion, Intimacy* × *Commitment*, and *Intimacy* × *Passion* × *Commitment*) to examine if different kinds of love as per Sternberg's theory are differently related to reproductive success. Having in mind relatively limited sample size in this investigation, overly complex regression models might be underpowered to detect small effect size, but we tested this suggestion in a separate regression model. We created a model starting with control variables (age, sex, and BMI), with the love components (intimacy, passion, and commitment) in the second step, and in the final, third step, we proceeded with interaction terms. The interaction terms did not improve the model (Δ*R*^2^ = 0.003, *p* value for change = 0.87) and none of the introduced cross-products was significant (all *p*s > 0.42) neither in general model nor in specific models created for men and women; thus, we restrained from further tests with this regard. We do highlight, however, that future investigations using larger samples and person-centered analytical approaches (e.g., latent profile analysis) might benefit from such an approach.

## Appendix

1. __ is able to count on me in times of need.
2. I am willing to share myself and my possessions with __.
3. I give considerable emotional support to __.
4. I feel that I can really trust __.
5. I share deeply personal information about myself with __.
6. I find myself thinking about __ frequently during the day.
7. I find ___ to be very personally attractive.
8. I would rather be with __ than with anyone else.
9. There is nothing more important to me than my relationship with __.
10. I especially like physical contact with __.
11. I cannot imagine my life without __.
12. I know that I care about __.
13. Because of my commitment to __, I would not let other people come between us.
14. I will always have a strong responsibility for __.
15. I view my relationship with __ as permanent.
16. I plan to continue my relationship with ____________.
17. Even when __ is hard to deal with, I remain committed to our relationship.
